# Facile Synthesis of ZnO/WO_3_ Nanocomposite Porous Films for High-Performance Gas Sensing of Multiple VOCs

**DOI:** 10.3390/nano13040733

**Published:** 2023-02-15

**Authors:** Biao Lei, Hongwen Zhang, Qian Zhao, Weiwei Liu, Yi Wei, Yanyan Lu, Tingting Xiao, Jinglin Kong, Weiping Cai

**Affiliations:** 1 Key Lab of Materials Physics, Anhui Key Lab of Nanomaterials and Nanotechnology, Institute of Solid State Physics, HFIPS, Chinese Academy of Sciences, Hefei 230031, China; 2 Science Island Branch of Graduate School, University of Science and Technology of China, Hefei 230026, China; 3 Lu’an Branch, Anhui Institute of Innovation for Industrial Technology, Lu’an 237100, China; 4 State Key Laboratory of NBC Protection for Civilian, Beijing 102205, China

**Keywords:** gas sensing, VOC detection, ZnO/WO_3_ composite films, in situ fabrication

## Abstract

Volatile organic compounds (VOCs) in indoor environments have typical features of multiple components, high concentration, and long duration. The development of gas sensors with high sensitivity to multiple VOCs is of great significance to protect human health. Herein, we proposed a sensitive ZnO/WO_3_ composite chemi-resistive sensor facilely fabricated via a sacrificial template approach. Based on the transferable properties of self-assembled monolayer colloidal crystal (MCC) templates, two-dimensional honeycomb-like ordered porous ZnO/WO_3_ sensing matrixes were constructed in situ on commercial ceramic tube substrates with curved and rough surfaces. The nanocomposite thin films are about 250 nm in thickness with large-scale structural consistency and integrity, which facilitates characteristic responses with highly sensitivity and reliability. Furthermore, the nanocomposite sensor shows simultaneous responses to multiple VOCs that commonly exist in daily life with an obvious suppression sensing for traditional flammable gases. Particularly, a detection limit of 0.1 ppm with a second-level response/recovery time can be achieved, which is beneficial for real-time air quality assessments. We proposed a heterojunction-induced sensing enhancement mechanism for the ZnO/WO_3_ nanocomposite film in which the formation of abundant heterojunctions between ZnO and WO_3_ NPs significantly increases the thickness of the electron depletion layer in the bulk film and improves the formation of active oxygen species on the surface, which is conducive to enhanced responses for reducing VOC gases. This work not only provides a simple approach for the fabrication of high-performance gas sensors but also opens an achievable avenue for air quality assessment based on VOC concentration detection.

## 1. Introduction

Volatile organic compounds (VOCs) are mainly emitted from anthropogenic sources such as fossil fuel, paints, compressed aerosol, and biomass combustion, which have been recognized as critical precursors for tropospheric ozone and secondary organic aerosols (SOAs) [1,2,3,4]. Environmental problems generated by VOCs can also cause direct harm to respiratory, allergic, or immune systems of human beings, and even cancer in organs [5]. Nowadays, VOCs have been recognized as prominent hazards in the environmental atmosphere and have evolved into a global issue faced by all mankind [6]. Plenty of studies have shown that indoor building paints and household products can constantly release complex organic compounds, and their concentrations in indoor air may be an order of magnitude higher than those outdoors [7]. Obviously, this will cause longer and more serious damages to human health. Therefore, real-time monitoring of multiple VOCs and early warnings of air pollution have extreme significance in air quality assessment and human health protection [8]. At present, the identification and quantification of atmospheric VOCs mainly rely on analytical instruments such as gas chromatography (GC) and mass spectrometry (MS), which are commonly used for the accurate analysis of air components [9,10,11,12,13]. However, despite high accuracy and low detection limits, those spectroscopic methods usually require expensive instruments, complex sample handling, and long analysis periods, which are not conducive to frequent air quality assessments in daily life [14,15,16]. Therefore, it is urgent to develop a cost-efficient, real-time method for the sensitive detection of multiple VOCs simultaneously.

It has been well-recognized that gas sensors based on metal oxide semiconductors have the remarkable advantages of low cost, simple operation, and easy usage, and they have been widely applied in the field of leakage alarm for flammable and harmful gases [17,18,19]. It is known that more adsorption or reaction sites can be provided for nanomaterials due to their high specific surface area, which is beneficial to the development of high-performance devices [20,21,22]. For decades, many research groups have reported the trace detections of individual VOC targets (such as toluene, formaldehyde, ethanol, etc.) with diverse metal oxide nanostructures [23,24,25,26,27,28]. However, those works are commonly focused on improvements in the selectivity and sensitivity towards one single organic vapor, and inadvertently ignore or even deliberately reduce the response to other organic gases, which makes it difficult to achieve air quality assessment by monitoring multiple VOCs. Currently, investigations on the simultaneous sensing of diverse VOCs are also reported. For instance, Nguyen et al. synthesized hollow, cubic assembled nanocrystal Zn_2_SnO_4_ via the one-step hydrothermal method for the simultaneous detection of acetone and ethanol [29]. Vandna et al. prepared a Pt-sensitized MoO_3_/mpg-CN mesoporous nanohybrid for the detection of targeted VOCs of acetone, ethanol, toluene, and n-butanol [8]. However, some significant drawbacks still exist, such as low sensitivity and long response/recovery time. The main reason is that a single type of sensing oxide or traditional noble metal surface sensitization is extremely limited in improving the cross-sensitivity of organic vapors. Alternatively, it has been reported that the combination of two semiconductors is beneficial for adjusting the space-charge layer on the reactive surface of the sensing material, thereby improving the gas-sensing performance in terms of sensitivity and response time [30,31]. Obviously, the combination of two sensing oxides can also inherit the response characteristic towards specific gases of individual material, which is beneficial to expanding the types and quantities of target gases. However, it is still a challenge to simply combine two gas-sensing materials into one sensing matrix and effectively adjust their structure and composition parameters to optimize their sensing performance, especially to realize device construction on conventional ceramic tube substrates with rough and curved surfaces.

Herein, we proposed a template-assisted one-pot synthesis of ZnO/WO_3_ nanocomposite porous thin films, which possess two-dimensional (2D) ordered honeycomb-like structures with structural uniformity and integrity over the entire ceramic tube substrates. It demonstrates that the relative content of the two sensing oxides can be facilely adjusted as desired by simply changing the chemical composition of the utilized precursors. Furthermore, the ZnO/WO_3_ nanocomposite film with a 5% proportion of ZnO can sensitively respond to multiple VOCs that commonly exist in daily life (benzenes, aldehydes, alcohols, and ketones vapors) while suppressing other flammable or toxic gases. The detection limit can be down to 0.1 ppm with a second-order response, which facilitates the trace and real-time detection for air quality assessment. The proposed template-mediated in situ fabrication approach possesses wide versatility to diverse sensing substrates, such as curved ceramic tubes, interdigitated planar plates, and cantilever-equipped MEMS chips, which is beneficial to the innovative design of not only VOC sensors, but also broad sensing matrixes for gas detection applications.

## 2. Experimental Section

### 2.1. Materials

Ammonium metatungstate ((NH_4_)_6_H_2_W_12_O_40_·5H_2_O) and zinc acetate dihydrate ((CH_3_COO)_2_Zn·2H_2_O) were purchased from Sinopharm Chemical Reagent Co., Ltd. (Shanghai, China). Polystyrene (PS) sphere suspension (2.5 wt% in water, 500 nm in diameter) was obtained from Shanghai Huge Biotechnology Co., Ltd. (Shanghai, China). All the reagents were of analytical grade and used without further purification. Deionized water was produced in an ultrafilter system (Millipore Milli-Q system) with a resistivity of 18.2 MΩ·cm at 25 °C.

### 2.2. In Situ Fabrication of ZnO/WO_3_ Ordered Porous Films

**Figure 1:**
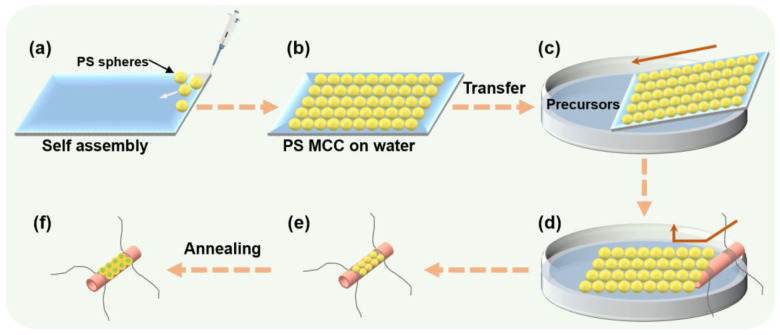
Schematic diagram of template-assisted in situ preparation of ZnO/WO_3_ nanocomposite porous sensing films. (**a**,**b**) A PS monolayer colloidal crystal (MCC) was prepared by interfacial self-assembly. (**c**–**e**) The MCC was transferred intact to the precursor surface, followed by another transfer using a ceramic tube substrate. (**f**) ZnO/WO_3_ nanocomposite porous films were obtained by heat treatment at 400 °C for 2 h.

Figure 1 schematically shows the direct fabrication of ZnO/WO_3_ nanocomposite sensing films on ceramic tube substrates via a sacrificial template approach. Typically, the well-dispersed PS spheres were spread on the glass slide and self-assembled into hexagonally ordered arrays (Figure 1a) [32]. After natural drying, the monolayer colloidal crystal (MCC) was entirely transferred to the surface of the desired precursor solution and then re-transferred to the ceramic tube surface (Figure 1b,c). (NH_4_)_6_H_2_W_12_O_40_·XH_2_O and (CH_3_COO)_2_Zn·2H_2_O were mixed and dissolved in 50 mL distilled water in different proportions and stirred with a magnetic rotor for 10 min to obtain a homogeneous solution of the precursor so that the required precursor could infiltrate into the void between adjacent PS spheres of the MCC. After annealing at 400 °C for 2 h, the ZnO/WO_3_ porous film was in situ obtained on the ceramic tube substrate due to the thermal decomposition of organic spheres and precursors (Figure 1d–f). By adjusting the relative content of (CH_3_COO)_2_Zn·2H_2_O, ZnO/WO_3_ porous films with Zn/W atomic percentages of 3%, 5% and 10% were prepared and labeled as 3% ZnO/WO_3_, 5% ZnO/WO_3_, and 10% ZnO/WO_3_, respectively. The pure WO_3_ ordered porous film was also prepared as a control.

### 2.3. Characterization

The phase and crystal structures of the products were determined by X-ray diffraction (XRD) patterns, which were recorded with the X-ray diffractometer (the Philips X’Pert) with a 1D array detector using Cu Kα1 radiation (λ = 1.5406 Å). The morphology and structure of the product were observed by field emission scanning electron microscope (FE-SEM, FEI Sirion 200) and transmission electron microscope (TEM, JEOL JEM-2100). High-resolution transmission electron microscopic (HR-TEM) images and selected-area electron diffraction (SAED) patterns were acquired on a JEOL JEM-2100 transmission electron microscope at an operating voltage of 200 kV. The energy-dispersive X-ray spectroscopy (EDS) spots pattern scanning analysis was recorded by the TEM attachment. The X-ray photoelectron spectroscopy (XPS) analyses were carried out on a photoelectron spectrometer (ESCALAB 250XI) operated at an acceleration voltage of 15 kV and a current of 10 mA, and the binding energy was calibrated with reference to the C 1s binding energy 284.8 eV.

### 2.4. Gas-Sensing Tests

The sensor characterization was conducted by a STP4 intelligent gas-sensing analysis system (Nanjing Wisens Co. Ltd., Nanjing, China). The sensing performance of the fabricated devices was monitored in a sealed gas-sensing chamber at room temperature (25 °C) and 60% RH (relative humidity). Firstly, we injected enough liquid volatile organic compound into a fixed volume glass container to make it volatilize long enough to reach its saturated vapor concentration. Subsequently, a certain amount of target gas was extracted from the glass container through a micro syringe, and then it was injected into the test chamber. According to the volume ratio between the injected gas and the test chamber, the actual concentration of target gas in the test chamber was calculated. To illustrate the chemical gas-sensing ability of the ZnO/WO_3_ films, we focused on four representative analytes: alcohols, aldehydes, benzenes, and ketones. These gases represent well-known toxic VOCs. The gas responses of the sensors are evaluated as *R_a_/R_g_*, where *R_a_* is the resistance of the sensor in air and *R_g_* is the resistance of the sensor in VOCs. The time taken by the sensors to achieve 90% of the total resistance change was defined as the response/recovery time (*t*) when exposed to the target gases or normal atmospheric environment.

## 3. Results and Discussion

Hexagonal packed MCC together with infiltrated precursor solutions were obtained on a curved ceramic tube by transfer of the MCC, which was self-assembled with PS spheres of 500 nm in diameter. After calcination, the bowl-shaped porous ordered ZnO/WO_3_ films were obtained in situ due to the spherical geometry of the monolayer PS spheres. Subsequently, the structure, morphology, composition, and gas-sensing performances of the ZnO/WO_3_ sensing films were systematically evaluated.

### 3.1. Structural and Morphological Characteristics

**Figure 2:**
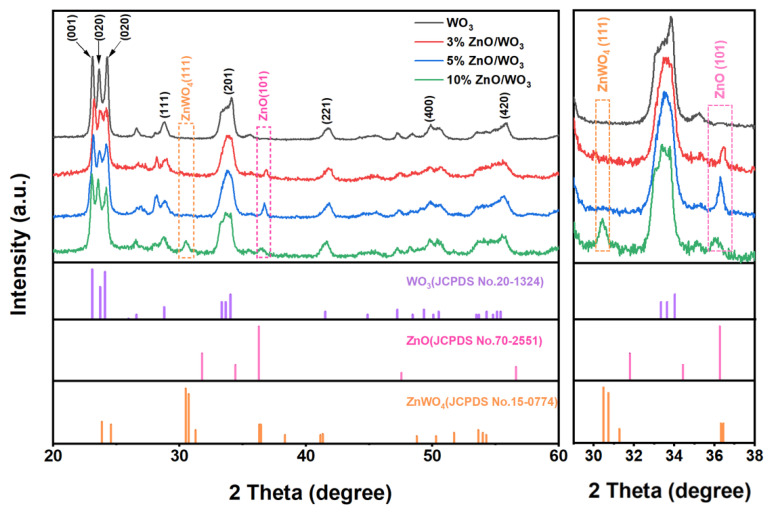
XRD patterns of the as-fabricated porous films with varied chemical compositions. The right figure is the enlargement of the diffraction area in the range of 29–38°, which clearly shows the detailed change of ZnO additive.

XRD analyses of the pristine WO_3_ and ZnO/WO_3_ nanocomposite films with different Zn/W atomic percentages were firstly conducted, as demonstrated in Figure 2. It reveals that the diffraction peaks of orthorhombic WO_3_ (JCPDS NO. 00-020-1324) were mainly observed at 2θ = 23.08°, 23.71°, and 24.10°, which were readily assigned to the (001), (020), and (200) lattice planes, respectively. After ZnO and WO_3_ were combined, the 3%, 5%, and 10% ZnO/WO_3_ films also showed additional peaks at 36.26°, arising from the diffraction of the (101) lattice plane of hexagonal ZnO (JCPDS NO. 01-070-2551). While there was an excessive combination of ZnO (10% ZnO/WO_3_), a monoclinic ZnWO_4_ (JCPDS NO. 00-015-0774) would be also obtained.

**Figure 3:**
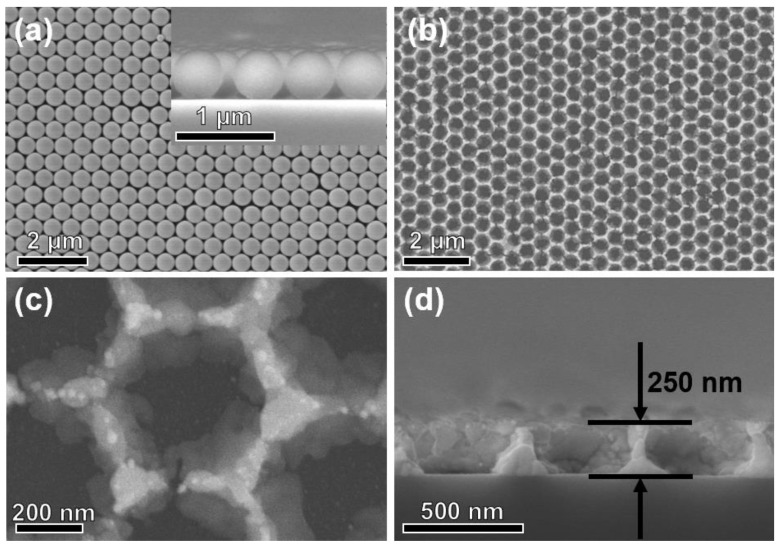
(**a**) FE-SEM view of the PS MCC template on a slide. The upper right illustration is the corresponding cross-section; the typical FE-SEM image of (**b**) the top view, (**c**) a local magnified view, and (**d**) a cross-section of 5% ZnO/WO_3_ porous film with 500 nm pore size.

Despite the differences in chemical composition, it is rational for the template-assisted method to produce metal oxide sensing films with similar microstructures. Therefore, the 5% ZnO/WO_3_ porous film was chosen for the following detailed demonstrations. Figure 3a shows the FE-SEM observation of the utilized MCC template prepared by the self-assembly process. It reveals that the PS spheres are hexagonally packed close to each other to form two-dimensional (2D) colloidal crystals with long-range order. The upper illustration of Figure 3a confirms its monolayer feature. After calcination in an air atmosphere, PS spheres were removed and the honeycomb ZnO/WO_3_ composite porous film was readily obtained (Figure 3b). The ZnO/WO_3_ nanocomposite sensing layer directly constructed on the substrate retains a large-scale ordered arrangement of nanopores, revealing a faultless template replication process. The porous structure makes the sensing layer have good mechanical stability. It is worth noting that the so-called “ordered porous” here refers to the overall honeycomb porous structure of the films, rather than the existence of many micropores or mesopores inside the films. The magnified observation (Figure 3c) illustrates that a single hexagonal aperture is about 500 nm. The monolayer structure is demonstrated by the cross-section SEM image in Figure 3d. After the annealing treatment to remove the PS template, the thickness of the films finally obtained is the radius size of the colloidal crystal (about 250 nm). Compared with the sensing films prepared by other brush-coated methods, the ultrathin sensitive films prepared by the in situ growth method are more uniform and stable, and the specific surface area is larger, which is conducive to the sensing response of gas. This ultra-thin, porous structure facilitates the diffusion of gas molecules from the surface to the interior of the film, thereby improving the sensing performance in terms of gas-sensitive response and response/recovery time.

**Figure 4:**
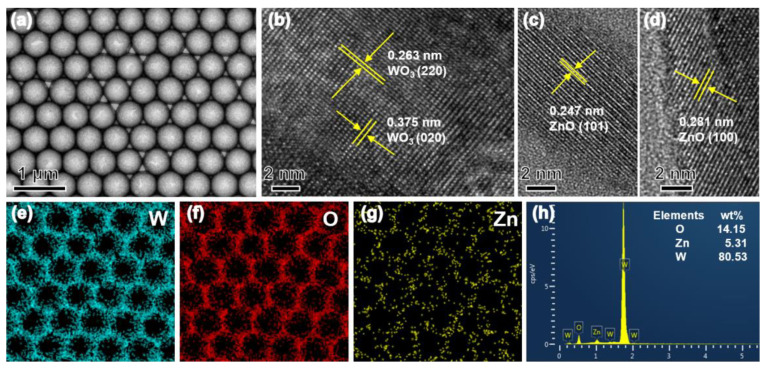
(**a**) Typical TEM observation of ZnO/WO_3_ sensing arrays fabricated in situ on ceramic tube clearly reveals the ordered arrangement ZnO/WO_3_ layers; (**b**–**d**) HR-TEM images; (**e**–**g**) elemental mapping images of ZnO/WO_3_ porous film; (**h**) the elemental analysis of energy-dispersive spectrometer (EDS) towards ZnO/WO_3_ film.

The morphology and crystallinity of the porous films were characterized using transmission electron microscopy (TEM) technologies. Figure 4a shows that the particle size of the large-scale 5% ZnO/WO_3_ porous film is 500 nm. In the imaging area, the whole porous film is tightly arranged with uniform pore size. Figure 4b–d show the high-resolution TEM (HR-TEM) images of WO_3_ and ZnO, respectively. The spacing between adjacent fringes is about 0.263 nm and 0.375 nm, which belong to the (220) and (020) crystal planes of WO_3_, while the spacing between the neighboring fringes is about 0.247 nm and 0.281 nm, belonging to the (101) and the (100) crystal planes of hexagonal ZnO phase. Moreover, based on the energy-dispersive X-ray spectroscopy (EDS) elemental analysis, the O, W, and Zn elements were uniformly dispersed on the surface of the 5% ZnO/WO_3_ porous film (Figure 4e–g). The EDS result shown in Figure 4h demonstrates that the peaks of O, Zn, and W can be clearly seen in the survey spectrum. The 5% ZnO/WO_3_ sensor was thus obtained.

**Figure 5:**
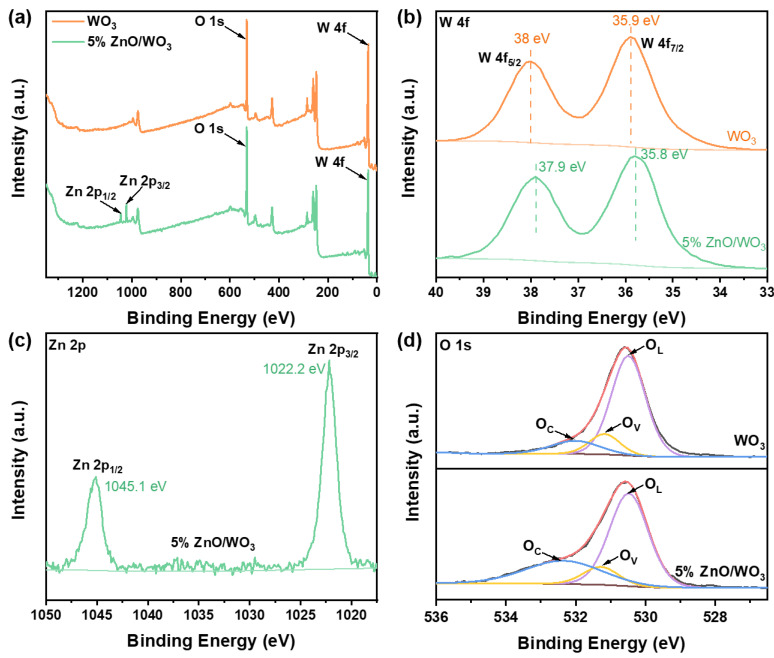
XPS spectra of WO_3_ and 5% ZnO/WO_3_ samples. (**a**) Survey spectrum and high-resolution spectra for (**b**) W 4f, (**c**) Zn 2p, and (**d**) O 1s.

In order to further investigate the elemental composition and chemical states of each element, X-ray photoelectron spectra (XPS) measurements were conducted for the WO_3_ and 5% ZnO/WO_3_ sensing films (Figure 5). They reveal that the WO_3_ film is only composed of two elements, W and O, while the 5% ZnO/WO_3_ film has an additional Zn element, which was consistent with the previous XRD and EDS results (Figure 5a). The high-resolution spectra of W 4f for both films (Figure 5b) show two distinct peaks located at 37.9 and 35.8 eV, which can be attributed to W 4f_5/2_ and 4f_7/2_, respectively, suggesting the presence of W^6+^ in the film matrixes [33]. In comparison with the W 4f spectrum of WO_3_, the 4f_5/2_ and 4f_7/2_ spin–orbit peaks of 5% ZnO/WO_3_ shift to lower binding energies of 37.9 and 35.8 eV, indicating that an n–n heterojunction might exist at the interface of ZnO and WO_3_, and a part of the electrons might transfer. Furthermore, the spectrum of Zn 2p for the 5% ZnO/WO_3_ film (Figure 5c) specifically shows two strong peaks located at 1045.1 and 1022.2 eV, which correspond to Zn 2p_1/2_ and 2p_3/2_ spin–orbit of the Zn^2+^ chemical state, respectively. Meanwhile, for the high-resolution O 1s spectra, the binding energies at 530.3, 531.3, and 532.4 eV can be ascribed to the lattice oxygen (O_L_), oxygen vacancy (O_V_), and chemisorption oxygen (O_C_), respectively [34]. O_L_ was commonly considered as a non-active oxygen species, which did not participate in the sensing reaction with target molecules, while O_V_ and O_C_ are positively related to the amount of active oxygen species adsorbed on the surfaces. The proportion of varying oxygen species for two sensing materials is displayed in Table 1, which displays that the addition of ZnO increases the content of O_V_ and O_C_ to 43%, thereby providing more potential to improve the gas-sensing performances for the 5% ZnO/WO_3_ sensors.

### 3.2. Gas-Sensing Performances

**Figure 6:**
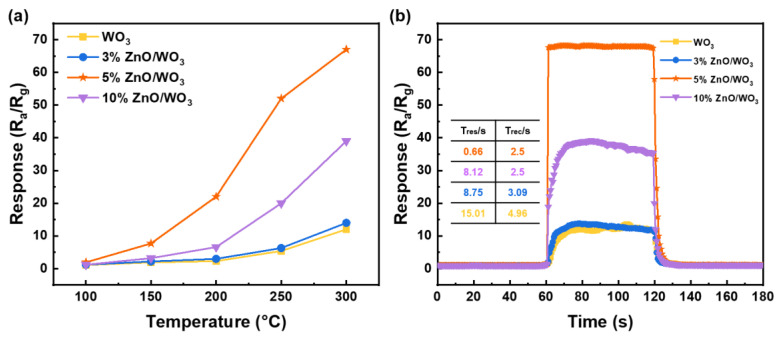
(**a**) Sensor responses to 100 ppm toluene vapor at operating temperatures of 100–300 °C. (**b**) Typical response curves for a single sensing cycle of 100 ppm toluene vapor at the optimal operating temperature of 300 °C.

The VOC gas-sensing properties of the four sensors were investigated in detail. Herein, we use toluene as a typical target gas to evaluate the gas-response performances. On account of the importance of working temperature for metal oxide semiconductor gas sensors, we tested the response of sensors at different temperatures to obtain the optimal operation temperature. Figure 6 illustrates the responses of these sensors to 100 ppm toluene vapor in the temperature range of 100–300 °C. The results show that all the responses of these four sensors are the highest at about 300 °C. Even as the sensors’ operating temperatures rise to 300 °C, their response tends to increase. This is consistent with that of traditional n-type semiconductor sensors for reducing gases. However, as continued temperature increase will result in a high energy consumption, which does not meet the actual real-time detection requirements, 300 °C is selected as the optimal operation temperature of those sensors in present work. In addition, the combination of ZnO and WO_3_ leads to significant enhancement in sensing responses to toluene compared with the pure WO_3_ sensor in the temperature range of 100 °C to 300 °C. Simultaneously, the 5% ZnO/WO_3_ sensor demonstrates the best response performance over all temperature ranges, and for excess addition of ZnO (the 10% ZnO/WO_3_ sensor), the gas-sensitive performances are remarkably decreased [30,35,36]. It can be seen from the XRD pattern (Figure 2) that a new material ZnWO_4_ appears in the 10% ZnO/WO_3_ composite system. The formation of ZnWO_4_ may be the main reason for the decrease of gas sensitivity of 10% ZnO/WO_3_. The gas sensitivity of the ZnO/WO_3_ composite system will be improved with the increase of ZnO content, and the gas sensitivity will reach the best when the molar content of ZnO is 5%. From the subsequent gas-sensing enhancement mechanism, we can know that there are n–n heterostructures in the ZnO/WO_3_ composite system, which will significantly improve the gas-sensing performance. The production of ZnWO_4_ will break the original best composite system, thus reducing the gas sensitivity of 10% ZnO/WO_3_. The same change trends can be observed for typical single-cycle response curves of the four sensors at the optimal operating temperature of 300 °C. This emphasizes the significant importance of the appropriate combination of ZnO for WO_3_ sensors. In addition, a response of *R_a_/R_g_* = 68 was obtained for the 5% ZnO/WO_3_ sensor towards 100 ppm toluene with a response and recovery time of 0.66 s and 2.5 s, respectively. An assessment of sensing performances towards toluene vapor in the literature is overviewed in Table 2, which confirms a significant advance of the as-prepared ZnO/WO_3_ sensor in present work. The response/recovery times of the ZnO/WO_3_ sensor for toluene are all less than 3 s, which is much less than that of other toluene-sensing materials [37,38,39]. Such sensitive and fast responses are beneficial to the trace and real-time detection of toxic VOCs for widespread household air quality-monitoring applications.

**Figure 7:**
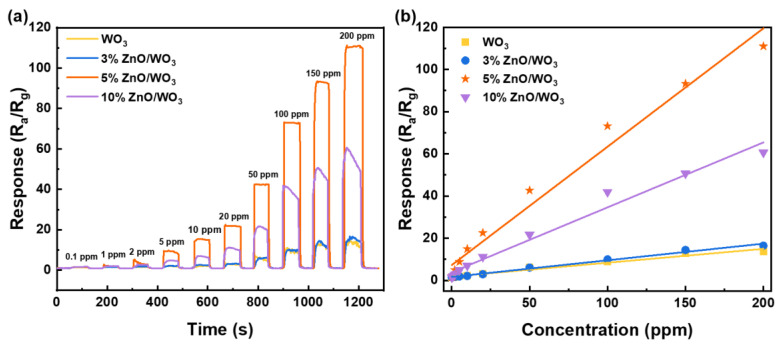
(**a**) Responses of the four sensors to toluene vapor with concentrations varying from 0.1 ppm to 200 ppm at the operating temperature of 300 °C. (**b**) Corresponding sensitivities as a function of the toluene vapor concentration.

The dynamic response–recovery curves of the four sensors to toluene vapor with stepped concentrations were systematically evaluated at 300 °C, as demonstrated in Figure 7a. Although the responses of all these gas sensors steadily increased with increasing toluene concentration, the 5% ZnO/WO_3_ sensor displayed the most significant sensitive performance. Despite having the strongest response to the target gas, the 5% ZnO/WO_3_ sensor showed much smoother fluctuations of the electrical signal at the equilibrium state, revealing a perfect signal stability. Moreover, the composite sensor had a limit of detection (LOD) down to 0.1 ppm, while pure WO_3_ had an LOD of 10 ppm. Meanwhile, it was difficult for other sensing materials (Table 2) to achieve such a low detection limit. The relationship between the response of the ZnO/WO_3_ sensor and the concentration of toluene was further studied. As exhibited in Figure 7b, the response values followed an approximately linear increase with the increase of toluene concentration in the range of 0.1−200 ppm, demonstrating that the 5% ZnO/WO_3_ sensor can work as a toluene vapor sensor in a wide linear range.

**Figure 8:**
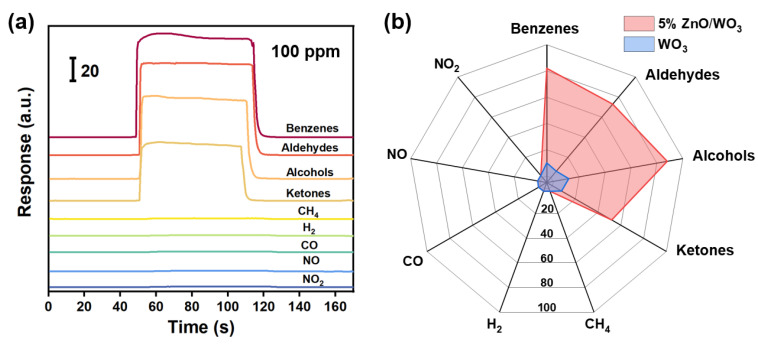
(**a**) Typical response curves of the 5% ZnO/WO_3_ sensor towards representative VOC targets (typically benzenes, aldehydes, alcohols, and ketones) and conventional flammable or toxic gases (methane, hydrogen, carbon oxide, nitric oxide, and nitric dioxide) with a concentration of 100 ppm at 300 °C. (**b**) Comparison of sensitivities of the WO_3_ and 5% ZnO/WO_3_ sensors to evaluate their sensing selectivity properties.

As we focus on assessing the air quality by monitoring concentrations of TVOC (total volatile organic compounds), it is expected that the sensor should have simultaneous response towards multiple organic solvents that commonly exist in daily life while suppressing the sensing performances to conventional flammable or toxic gases. Therefore, in addition to toluene, we also chose formaldehyde, ethanol, and acetone as representative VOC targets, and several reducing/oxidizing gas molecules such as methane, hydrogen, carbon oxide, nitric oxide, and nitric dioxide as interfering gases. Figure 8a demonstrates that the 5% ZnO/WO_3_ sensor shows sensitive and rapid response upon exposure of those individual VOC targets with concentrations of 100 ppm at 300 °C, while no obvious response can be observed for all those interfering gases. Furthermore, in comparison with the WO_3_ sensor, the 5% ZnO/WO_3_ sensor shows significant improvement in responses to those organic vapors, revealing a perfect cross-sensitivity towards VOC targets (Figure 8b). This feature facilitates accurate recognition of the characteristic gaseous constituents in complex environmental conditions, thereby providing more reliable diagnostic data for air quality assessment.

**Figure 9:**
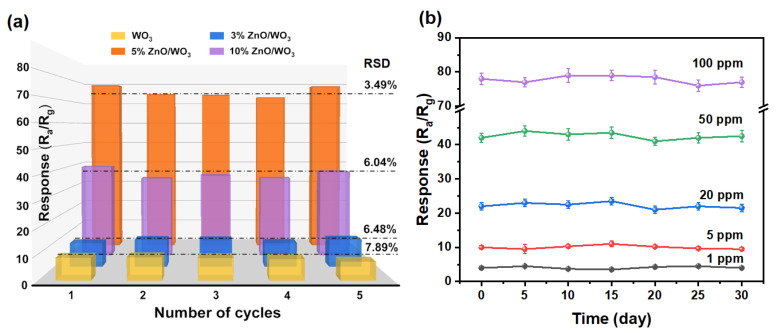
(**a**) Responses of the four sensors towards five repeatable circles towards 100 ppm toluene vapor at the operating temperature of 300 °C. (**b**) Long-term stability of 5% ZnO/WO_3_ films’ gas sensors to 1, 5, 20, 50, and 100 ppm toluene vapor.

Sensing stability is one of the key criteria to evaluate whether the gas sensor can be used for practical applications. A good stability requires that the sensor can repeatedly respond to the target gas of a specific concentration without significant response decreases. Hence, the response reproducibility of the four sensors to repeated exposure of toluene vapor were experimentally explored. Figure 9a demonstrates typical sensing responses for five repeatable circles to 100 ppm toluene vapor at the operating temperature of 300 °C. It depicts that although the RSD (relative standard deviation) values of the corresponding responses were all less than 10%, the 5% ZnO/WO_3_ sensor showed the best signal reproducibility (RSD = 3.49%) among the four sensors, which is of great significance for reliable quantitative gas monitoring. Furthermore, the long-term stability of the 5% ZnO/WO_3_ sensor to toluene with different concentrations was also measured, as demonstrated in Figure 9b. It reveals that the sensor exhibited nearly constant responses (RSD < 2%) to 1, 5, 20, 50, and 100 ppm toluene with a long period of intermittent tests, confirming a perfect stability of the as-developed ZnO/WO_3_ sensors for VOC detection.

### 3.3. Gas-Sensing Mechanism

**Figure 10:**
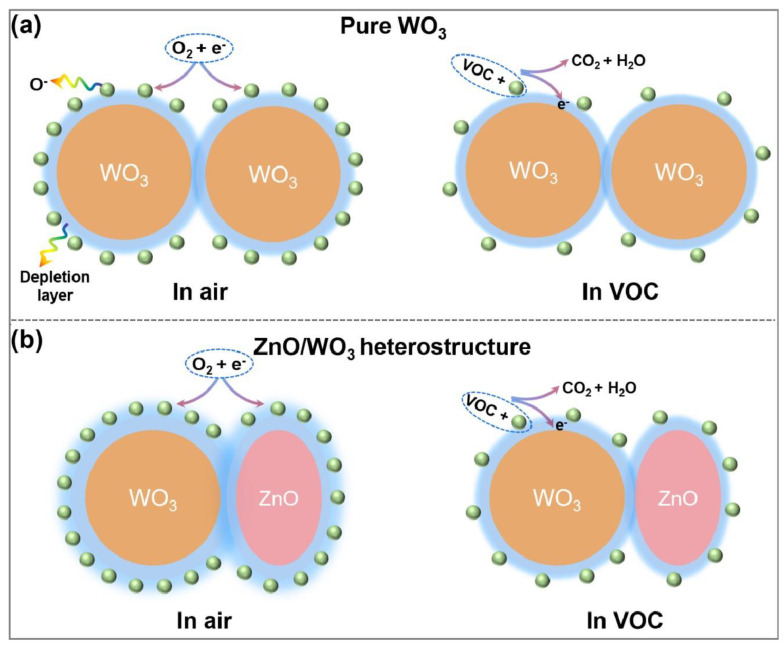
Schematic gas sensing diagram of (**a**) pure WO_3_^−^ and (**b**) ZnO/WO_3_ nanocomposite-based sensors exposed to air and VOC, respectively. The space-charge layer model is used to explain the sensing mechanism.

Gas sensing is known to be directly related to the adsorption of active species and subsequent surface reactions [30]. At present, the most widely accepted sensing mechanism in the literature is based on the redox reaction between the pre-adsorbed oxygen species from the environment and the following target molecules, which occurs on the surface of sensing materials [44,45,46]. When an n-type semiconducting matrix is exposed to the air (Figure 10a), oxygen molecules are adsorbed on the surface and subsequently capture free electrons from the conductance bands of the semiconductor, forming negatively charged surface oxygen species (O_2_^−^, O^−^, O^2−^) [36]. As a result, an electron depletion layer (EDL) forms on the surface domains, and the sensing matrix is in a high-resistance state. The corresponding reaction processes are shown in Equations (1)–(4) [47]:

(1) $$O_{2}\left(gas\right)↔O_{2}\left(ads\right)$$

(2) $$O_{2}\left(ads\right)+e^{-}↔O_{2}^{-}\;(T\;<\;100\;{}^{\circ}C)$$

(3) $$O_{2}^{-}\left(ads\right)+e^{-}↔2O^{-}\;(100\;{}^{\circ}C\;\leq \;T\;\leq \;300\;{}^{\circ}C)$$

(4) $$O^{-}\left(ads\right)+e^{-}↔O^{2-}\;(T\;>\;300\;{}^{\circ}C)$$

Furthermore, it has been reported that the formation of heterogeneous structures between two metal oxide semiconductors can significantly improve the adsorption of O_2_ due to the existence of electronic effects [35,48,49]. Thus, more O_2_ adsorption sites exist on the surface of ZnO/WO_3_ nanocomposite films due to the formed n–n heterojunction. Accordingly, compared with pure WO_3_, the ZnO/WO_3_ composite possesses a thicker depletion layer on the surface and produces a larger bulk resistance (Figure 10b and Figure S1). When the sensing matrix is exposed to the reducing toluene vapor, the toluene molecules undergo a redox reaction with the surface-adsorbed oxygen species and release electrons into the interior of sensing material. For the ZnO/WO_3_ composite, it will result in a more significant decrease in the bulk resistance and more active oxygen species on the surface, which produce enhanced gas-sensing properties [50].

## 4. Conclusions

In summary, we proposed a sacrificial template-based in situ approach for the facile fabrication of ZnO/WO_3_ ordered nanoporous sensing films on commercial ceramic tubes with curved and rough surfaces. The as-fabricated nanocomposite films possess two-dimensional honeycomb-like porous features with 250 nm in thickness and large-scale structural consistency. Such features endue the nanocomposite sensors with sensitive responses, fast response/recovery, and repeatable detection performances. Typically, for the 5% ZnO/WO_3_ composite sensor, a detectable concentration of 0.1 ppm with a second-level response time and perfect signal reproducibility (RSD = 3.49%) was obtained. Furthermore, we verified that the combination of two sensitive materials would generate abundant heterojunctions inside and on the surfaces of the sensing matrixes. This significantly increases the thickness of the charge depletion layer and the adsorption of active oxygen species on the surface, which is conducive to increasing the cross-sensitivity of the gas sensor to VOCs while suppressing the response to other kinds of interfering gases. The proposed in situ strategy for the preparation of nanocomposite sensing films is not only conducive to optimizing the performance of gas sensors through the regulation of nanostructures and chemical composition, but also provides a technical route for the development of cross-sensitive gas sensors.

## Figures and Tables

**Table 1:** The content ratio of oxygen species derived from the fitting curves of the O 1s XPS spectra of WO_3_ and 5% ZnO/WO_3_ sensing matrixes.

Samples	O_L_ (eV)	O_V_ (eV)	O_C_ (eV)	O_L_ (%)	O_V_ (%)	O_C_ (%)	O_V_ + O_C_ (%)
**WO_3_**	530.3	531.2	532.1	73	13	14	27
**5% ZnO/WO_3_**	530.3	531.3	532.4	57	14	29	43

**Table 2:** Comparison of the sensing performances for the present 5% ZnO/WO_3_ sensor for toluene vapor with those reported in the literature.

Sensing Materials	Con. (ppm)	Tem. (°C)	Response (Ra/Rg)	Res./Rec. Time (s)	Detection Limit (ppm)	Ref.
Zn_2_SnO_4_ sheet	100	280	25.2	1/3.5	5	[26]
Au-ZnO-NP	100	377	97	65/360	/	[37]
Ni–ZnO core–shell spheres	100	325	210	2/77	0.5	[38]
Au-functionalized WO_3_·H_2_O	100	300	50	2/9	/	[40]
Pd-NPs/Pd-embedded WO_3_ NFs	5	350	10	10.9/16.1	0.2	[41]
Core–shell ZnFe_2_O_4_ spheres	100	275	55.26	3/105	0.2	[39]
WO_3_ microflowers	100	320	16.7	2/12	1	[42]
Hierarchical Au-loaded WO_3_ hollow microspheres	100	340	24	8/5	5	[43]
ZnO/WO_3_ composite ordered porous films	50	300	68	0.7/2.5	0.1	This work

## Data Availability

No new data were created or analyzed in this study. Data sharing is not applicable to this article.

## Supplementary Materials

The following supporting information can be downloaded at: https://www.mdpi.com/article/10.3390/nano13040733/s1, Figure S1: The initial resistance of the four sensors in air atmosphere at 300 °C.
