# Supplementary material for: Facile Synthesis of ZnO/WO3 Nanocomposite Porous Films for High-Performance Gas Sensing of Multiple VOCs
Source: Nanomaterials (Basel). 2023 Feb 15;13(4):733. doi: 10.3390/nano13040733 (PMC9965940; doi:10.3390/nano13040733)
Supplement: Supplementary file 1 [file nanomaterials-13-00733-s001.zip › nanomaterials-2176152-supplementary.pdf]

Supporting Information

Facile Synthesis of ZnO/WO_3 Nanocomposite Porous Films for High Performance Gas Sensing to Multiple VOCs

Biao Lei,[1,2] Hongwen Zhang,[1,3*] Qian Zhao,[1] Weiwei Liu,[4] Yi Wei, [1,2] Yanyan Lu, [1,2] Tingting Xiao,[1] Jinglin Kong,[4*] and Weiping Cai [1,2*]

[1] *Key Lab of Materials Physics, Anhui Key Lab of Nanomaterials and Nanotechnology, Institute of Solid State Physics, HFIPS, Chinese Academy of Sciences, Hefei 230031, P.R. China*

[2] *University of Science and Technology of China, Hefei 230026, P.R. China*

[3] *Lu'an Branch, Anhui Institute of Innovation for Industrial Technology, Lu'an 237100, P.R. China*

[4] *State Key Laboratory of NBC Protection for Civilian, Beijing 102205, China*

[*] Address correspondence to hwzhang@issp.ac.cn, jlkong@sina.com, wpcai@issp.ac.cn

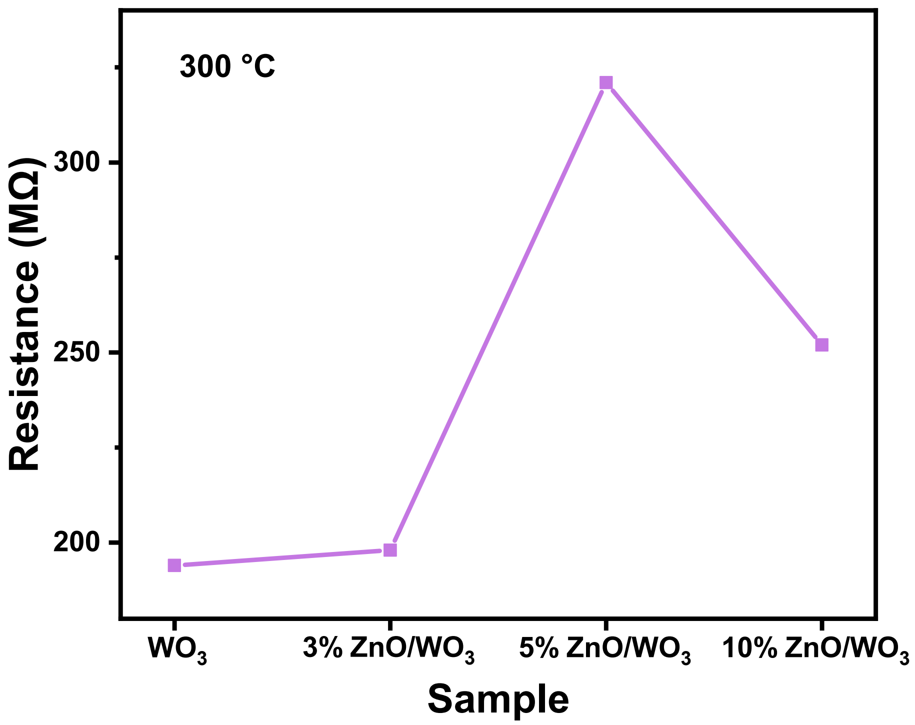

Figure S1. The initial resistance of the four sensors in air atmosphere at 300 ℃.
